# EFMlrs: a Python package for elementary flux mode enumeration via lexicographic reverse search

**DOI:** 10.1186/s12859-021-04417-9

**Published:** 2021-11-10

**Authors:** Bianca A Buchner, Jürgen Zanghellini

**Affiliations:** 1 Department of Biotechnology, University of Natural Resources and Life Sciences, Vienna, Austria; 2 Austrian Centre of Industrial Biotechnology, Vienna, Austria; 3 Department of Analytical Chemistry, University of Vienna, Vienna, Austria

**Keywords:** Elementary modes, Cobrapy, Metabolic modelling, Mplrs, Lexicographic reverse search, Systems biology

## Abstract

**Background:**

Elementary flux mode (EFM) analysis is a well-established, yet computationally challenging approach to characterize metabolic networks. Standard algorithms require huge amounts of memory and lack scalability which limits their application to single servers and consequently limits a comprehensive analysis to medium-scale networks. Recently, Avis et al. developed mplrs—a parallel version of the lexicographic reverse search (lrs) algorithm, which, in principle, enables an EFM analysis on high-performance computing environments (Avis and Jordan. mplrs: a scalable parallel vertex/facet enumeration code. arXiv:1511.06487, 2017). Here we test its applicability for EFM enumeration.

**Results:**

We developed EFMlrs, a Python package that gives users access to the enumeration capabilities of mplrs. EFMlrs uses *COBRApy* to process metabolic models from sbml files, performs loss-free compressions of the stoichiometric matrix, and generates suitable inputs for mplrs as well as efmtool, providing support not only for our proposed new method for EFM enumeration but also for already established tools. By leveraging *COBRApy*, EFMlrs also allows the application of additional reaction boundaries and seamlessly integrates into existing workflows.

**Conclusion:**

We show that due to mplrs’s properties, the algorithm is perfectly suited for high-performance computing (HPC) and thus offers new possibilities for the unbiased analysis of substantially larger metabolic models via EFM analyses. EFMlrs is an open-source program that comes together with a designated workflow and can be easily installed via pip.

**Supplementary Information:**

The online version contains supplementary material available at 10.1186/s12859-021-04417-9.

## Introduction

Arguably one of the most successful approaches in systems biotechnology and metabolic engineering are constraint-based methods (CBMs). These methods reconstruct (genome-scale) metabolic networks from genetic information and combine it with steady-state analysis to predict phenotypes from genotypes [2]. The success of CBMs is owed to the wealth of available metabolic information. Importantly, CBMs do not require any kinetic data as they focus on a steady-state description. In particular linear programming-based flux balance analysis (FBA) approaches have proven useful and scalable. Yet, FBA is biased as it selects the solution based on the optimal performance of an (selected) objective function. In fact, FBA characterizes optimal modes of operation rather than the available solution space.

In contrast, EFM analysis allows an unbiased characterization of a metabolic network, as it describes all feasible steady-state phenotypes in terms of elementary pathways, so-called EFMs, without the necessity of an optimality criterion [3]. EFMs are (support) minimal sets of reactions that can operate at steady-state while using all irreversible reactions in the appropriate direction [4]. The minimality property means that no reaction can be removed from the set of flux-carrying reactions without losing the ability to keep up a non-zero steady-state flux.

However, this definition of EFMs allows only two homogeneous inequality constraints - the steady-state assumption and the sign restrictions on the rates of irreversible reactions. Thus, a more general definition that also allows the incorporation of other inhomogeneous linear constraints, such as upper and lower reaction bounds, was needed. In 2007 Urbanczik et al. presented such a concept for the first time and expanded the definition of EFMs by introducing elementary flux vectors (EFVs) [5]. Although this concept initially received little attention, over the years it has been taken up again and further explored [6–8].

In further course we use the definition as proposed by Klamt et al. in 2017. It is an equivalent but more general definition of EFMs that also includes EFVs, by specifying EFMs as convex-conformally non-decomposable pathways [8] in a metabolic network. The latter definition also allows including inhomogeneous flux bounds into the analysis. Therefore, biologically, EFMs/EFVs (EFM/Vs) represent potential functional units in metabolic networks. In fact, every steady-state flux can be represented as a convex combination of its EFVs plus a conical linear combination of its EFMs [9, 10]. These properties make EFM analysis a powerful tool in basic biological research and metabolic engineering.

However, the enumeration of EFM/Vs is challenging, as the numbers of EFM/Vs grow combinatorially with the size of the metabolic network [11], which essentially limits the applicability of EFM analysis to small or medium size metabolic networks. Standard tools, e.g efmtool [12] or the FluxModeCalculator (FMC) [13], are difficult to parallelize and suffer from exorbitant memory consumption. Alternatives, e.g. Song et al. [14], Pey et al. [15] or Marashi et al. [16], use optimization principles to sequentially enumerate EFMs. In general, the latter are much slower than the former and only allow one to sample a subset of EFMs. Thus, the complete enumeration of EFM/Vs in genome-scale metabolic models remains intractable with current approaches [17].

Mathematically, the enumeration of EFM/Vs in metabolic networks is a vertex enumeration problem in convex polyhedra. There are essentially two approaches to solve this problem: (i) reverse search [18], and (ii) double description [19]. The former is typically considered unsuitable for enumerating vertices in highly-degenerate networks [20], such as metabolic networks. Recently, this assumption has been challenged by a multi-threading, parallelized version of the lrs algorithm [21]—mplrs [1]. Performance tests of this algorithm indicate that it is almost embarrassingly parallel and best suited for HPC due to its excellent scalability and negligible memory consumption. Here, we test the suitability of mplrs [1] for EFM enumeration in metabolic networks.

## Methods

### Mathematical representation and its geometric interpretation

In the following and without loss of generality, we assume that all variables unconstrained in sign are replaced by the difference of two non-negative variables. Thus, all variables are non-negative. In metabolic terms, this means that every reversible reaction was replaced by two counteracting irreversible reactions.

A convex polyhedron *P* is defined as the set of solutions to a system of linear inequalities

1 $$\begin{aligned} P= \{\varvec{x}\in {\mathbb {R}}^l \mid \varvec{A}\varvec{x}\ge \varvec{b}\}, \end{aligned}$$

with a matrix $\varvec{A}\in {\mathbb {R}}^{k\times l}$ and a vector $\varvec{b}\in {\mathbb {R}}^{k}$. Geometrically, *P* can be thought of as an intersection of half-spaces. As we assume $\varvec{x}\ge \mathbf{0 }$, (1) describes a pointed convex polyhedron sitting in the non-negative orthant.

Any convex polyhedron can be represented not only as an intersection of half-spaces [as in (1)], but also as a (Minkowski) sum of a “bounded” polytope and an “unbounded” cone

2 $$\begin{aligned} P= {{\,\mathrm{conv}\,}}(\varvec{\varepsilon }^1,...,\varvec{\varepsilon }^m) + {{\,\mathrm{cone}\,}}(\varvec{e}^1,...,\varvec{e}^n), \end{aligned}$$

where

3 $$\begin{aligned} {{\,\mathrm{conv}\,}}(\varvec{\varepsilon }^1,...,\varvec{\varepsilon }^m)&= \left\{ \sum _i \alpha _i\varvec{\varepsilon }^i \left| \sum _i\alpha _i=1, \alpha _i\ge 0 \right. \right\} , \end{aligned}$$

and

4 $$\begin{aligned} {{\,\mathrm{cone}\,}}(\varvec{e}^1,...,\varvec{e}^n)&= \left\{ \left. \sum _j \beta _j\varvec{e}^j \right| \beta _j\ge 0 \right\} \end{aligned}$$

denote polytope and cone spanned by the convex and conic combination of all extreme points, $\varvec{\varepsilon }^i$, and extreme rays, $\varvec{e}^j$, of the convex polyhedron *P*, respectively. Here, “extreme ray” means that not only the point $\varvec{e}^j$ is extreme and part of the convex polyhedron *P*, but all points sitting on the ray $\{\alpha \varvec{e}|\alpha \ge 0\}$ are too.

In the following, it will prove useful to explicitly collect equalities, inequalities, and state non-negativity in corresponding matrices and vectors and write (1) as

5 $$\begin{aligned} P= \{\varvec{x}\in {\mathbb {R}}^l \mid \varvec{C}\varvec{x}= \varvec{d}, \, \varvec{E}\varvec{x}\ge \varvec{f}, \, \varvec{I}\varvec{x}\ge \mathbf{0 }\}, \end{aligned}$$

with $\varvec{C}\in {\mathbb {R}}^{k_1\times l}$, $\varvec{d}\in {\mathbb {R}}^{k_1}$, $\varvec{E}\in {\mathbb {R}}^{k_2\times l}$, $\varvec{f}\in {\mathbb {R}}^{k_2}$, and an $l \times l$ identity matrix $\varvec{I}$. By setting

6 $$\begin{aligned} \varvec{A}= \begin{pmatrix}\varvec{C}\\ -\varvec{C}\\ \varvec{E}\\ \varvec{I}\end{pmatrix}, \text { and } \varvec{b}= \begin{pmatrix}\varvec{d}\\ -\varvec{d}\\ \varvec{f}\\ \mathbf{0 }\end{pmatrix}, \end{aligned}$$

the representation in (1) is recovered.

In general, (5) describes a convex polyhedron. We can transform this polyhedron into a convex polyhedral cone *PC* by embedding the polyhedron into a higher dimensional space

7 $$\begin{aligned} PC= \left\{ \varvec{y}\in {\mathbb {R}}^{l+1} \mid \varvec{G}\varvec{y}= \mathbf{0 }, \, \varvec{H}\varvec{y}\ge \mathbf{0 }\right\} , \end{aligned}$$

with

8 $$\begin{aligned} \varvec{G}= \begin{pmatrix}\varvec{C}&-\varvec{d}\end{pmatrix},\ \varvec{H}= \begin{pmatrix}\varvec{E}&{} -\varvec{f}\\ \varvec{I}&{} \mathbf{0 }\end{pmatrix},\ \varvec{y}= \begin{pmatrix}\varvec{x}\\ \zeta \end{pmatrix}, \end{aligned}$$

where we have introduced a new slack variable $\zeta$, which for $\zeta =1$ returns (5). Thus, it is possible to transform every polyhedron, specially every (“bounded”) polytope, into an (“unbounded”) convex cone that is embedded in a higher dimensional space. By doing so, every vertex enumeration problem can also be understood as an extreme ray enumeration problem.

In metabolic pathway analysis, we encounter a special convex polyhedron, referred to as flux cone

9 $$\begin{aligned} FC= \{\varvec{r}\in {\mathbb {R}}^n \mid \varvec{N}\varvec{r}= \mathbf{0 }, \, \varvec{I}\varvec{r}\ge \mathbf{0 }\}. \end{aligned}$$

Here, $\varvec{N}\in {\mathbb {R}}^{m\times n}$ and $\varvec{I}$ denote the stoichiometric matrix of the metabolic network and the $n\times n$ unity matrix, respectively, and $\varvec{r}\in {\mathbb {R}}^n$ denotes the vector of the flux distribution through the metabolic network. Biologically, $\varvec{N}\varvec{r}= \mathbf{0 }$ and $\varvec{I}\varvec{r}\ge \mathbf{0 }$ encode the steady-state condition and the irreversibilities of the reaction fluxes, respectively.

Clearly, by setting

10 $$\begin{aligned} \varvec{C}= \varvec{N}, \, \varvec{E}= \mathbf{0 }, \, \varvec{d}=\mathbf{0 },\, \varvec{f}= \mathbf{0 }, \, \text { and } \varvec{x}= \varvec{r}, \end{aligned}$$

(9) is a special case of (5). Conversely, by setting

11 $$\begin{aligned} \varvec{N}= \begin{pmatrix} \varvec{C}&{} \mathbf{0 }&{} -\varvec{d}\\ \varvec{E}&{} -\varvec{I}&{} -\varvec{f}\end{pmatrix}, \text { and } \varvec{r}= \begin{pmatrix}\varvec{x}\\ \varvec{\xi }\\ \zeta \end{pmatrix}, \end{aligned}$$

and using the slack variables $\varvec{\xi }\in {\mathbb {R}}^{k_2}_{\ge 0}$ and $\zeta \in {\mathbb {R}}_{\ge 0}$ we transform the polyhedron (5) into a flux cone (9).

In metabolic terms, the extreme rays of the flux cone (9) correspond to EFMs, if we disregard “two-cycle modes” that consist of the forward reaction and backward reaction of an originally reversible reaction.

Additionally, we can “read” the polyhedron (5) metabolically. For $\varvec{d}=\mathbf{0 }$ and $\varvec{x}=\varvec{r}$, we can interpret $\varvec{C}$ as a stoichiometric matrix, $\varvec{E}\varvec{x}\ge \varvec{f}$ as allocation and capacity constraints, and $\varvec{I}\varvec{x}\ge \mathbf{0 }$ as irreversibly constraints on the reaction fluxes. Except for two-cycle modes (see above), the extreme points and extreme rays of this polyhedron are then the EFVs and EFMs of the metabolic network.

Similarly, every element of a pointed flux cone can be represented as a conical combination of its extreme rays, which correspond to EFMs (if we again disregard ”two-cycle modes”). Thus, we can understand any flux distribution as a superposition of elementary metabolic units.

Both, EFMs and EFVs can be computed with mplrs [1] using either the formulations (1), (5), (7), or (9). However, efmtool [12] can only enumerate EFMs of the flux cone (9) making a transformation according to (6) necessary.

### Algorithms for calculating EFMs

#### Double description method

**Fig. 1:**
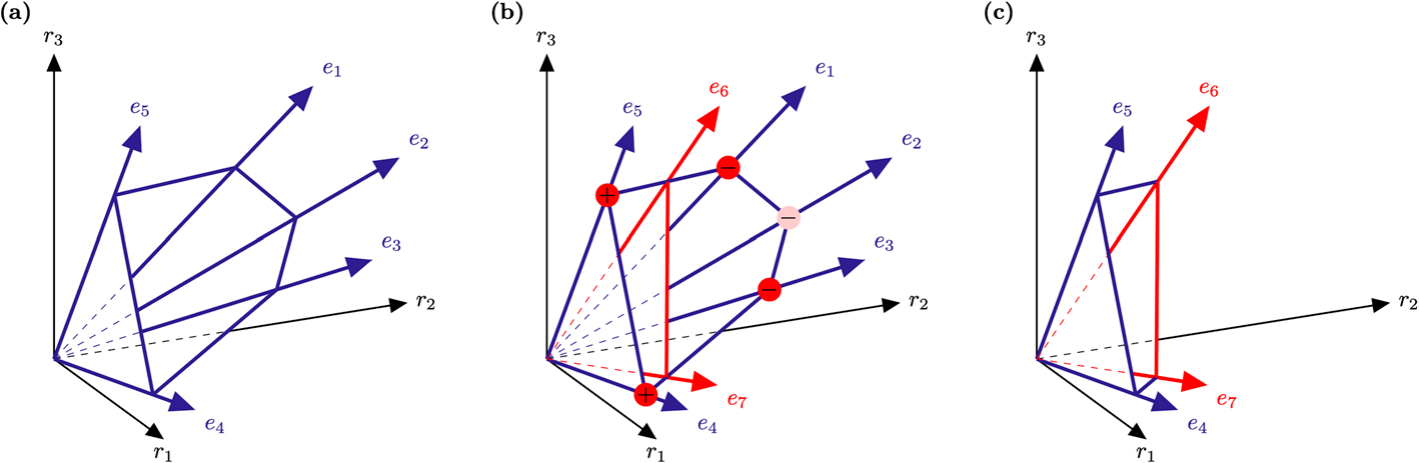
Iteration step of the DDM method visualizing the generation of a new set of extreme rays. **a** shows a cone with the rays $e_1$ to $e_5$. **b** shows the creation of a new set of extreme rays ($e_6$ and $e_7$) through hyperplane intersection and the pairwise combination of adjacent rays e.g. $e_5$ and $e_1$ result in the new extreme ray $e_6$, $e_4$ and $e_3$ in $e_7$. Other combinations e.g $e_5$ and $e_2$ are super-sets of $e_6$ and $e_7$ and do not result in new extreme rays. **c** shows the final cone with all redundant rays removed thus it only consists of the *actual* EFMs $e_4$, $e_5$, $e_6$ and $e_7$

The most commonly used method for the complete enumeration of EFMs in metabolic networks is the double description method (DDM).

efmtool uses the binary null-space implementation [22] of the DDM [23] to enumerate the “edges”, i.e. extreme rays, of a pointed flux cone. The algorithm starts from the kernel of the stoichiometric matrix *N* and iteratively converts it to binary form. For each reaction (i.e. in each row of the matrix) every column with a negative value at this reaction is replaced by all possible combinations of pairs of columns such that their conic sum is zero (Fig. 1). Eventually, the currently processed row of the such augmented matrix contains only non-negative numbers, which can be binarized. Some of the newly added columns may contain redundant information if they are super-sets of other columns and thus can be removed. For instance, the combinations $(e_2,e_4)$ and $(e_2,e_5)$ in Fig. 1b are super-sets of the new rays $e_6$ and $e_7$. The iteration stops if all reactions are processed and the resulting matrix contains all (binarized) EFMs in its columns.

During the iteration phase many candidate EFMs (i.e. columns in the matrix) are constructed that are not present in the final list of EFMs. For instance, the intermediate EFMs $e_1$ to $e_3$ in Fig. 1 are only required to construct $e_6$ and $e_7$. Thus, computational time and resources are used on calculating EFM *candidates* instead of *actual* EFMs. This is a major limitation of the DDM as due to this ex-post validation procedure it needs to keep all intermediate results in memory. Therefore, DDM requires a huge amount of random access memory (RAM) which for single core as well as for parallelized implementations, has to be available on one single server. Although, the required memory can be stored in an *out-of-core* memory instead as well, this approach is not computationally performative and was therefore disregarded. Hence, the RAM requirements of the DDM can easily exhaust even state-of-the-art systems [19]. Additionally, the unknown complexity of the DDM poses another obstacle since the required run time cannot be estimated from the input data.

Through parallelization and optimization techniques efmtool [12, 24] is one of the fastest implementations of the DDM. As efmtool runs on a Java virtual machine it is easily accessible and thus one of the most popular and commonly used implementations. Although FMC [13] is a more efficient implementation of the binary null-space DDM, it runs MATLAB and requires a paid license. Therefore, we decided to include support for efmtool in EFMlrs and use it as the representative of the DDM for comparisons with mplrs [1].

#### Lexicographic reverse search

mplrs [1] is an improved and parallel implementation of the reverse search algorithm [18]. It first finds some extreme point (thus the polyhedron needs to be pointed) and then systematically traces the polyhedron until no new extreme point or extreme ray can be found. It has been shown that this is in principle possible [25]. In fact, reverse search works by “inverting” the simplex algorithm for linear programming. The simplex algorithm first finds an extreme point (a basic feasible solution) and then moves along an edge of the polyhedron to an adjacent vertex, where the objective function has a greater value. This continues until the optimum (if it exists) is reached. With an appropriate pivot (i.e. a rule to pick an adjacent vertex) a unique path from any starting vertex to the optimum can be guaranteed. Collecting all these paths from all vertices generates a spanning tree [26] rooted at the optimum vertex. Reverse search starts at an extreme point, finds an objective function that makes this starting point optimal and maps out the spanning tree in depth-first order [18].

**Fig. 2:**
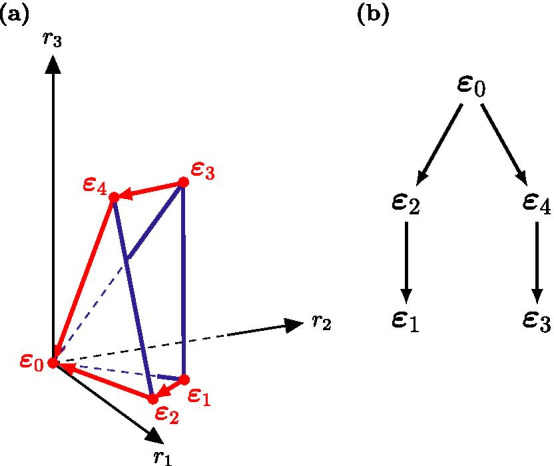
Path of the simplex method through a simple tetrahedron calculated with lrs [21]. For better readability we transformed the cone from Fig. 1 into a tetrahedron and only highlighted the path of the simplex method. **a** shows the path and direction of the simplex method indicated by the red arrows. **b** shows the corresponding reverse search tree. The black arrows indicate the path of the reverse search which starts at the origin vertex $\varvec{\varepsilon }_0$ and results in a search tree with a depth of 2

Figure 2 shows the path of the simplex algorithm for a *simple* tetrahedron. A polyhedron is regarded as *simple* or non-degenerate if each vertex lies on exactly *l* hyper-planes and therefore has a unique base. For such polyhedra, lrs is very efficient as the resulting spanning forest has a single component such that each vertex is produced once [21]. For these *simple* polyhedra, the run time only depends on the input data [1]. Yet, the flux cones of metabolic models are highly degenerate which is the main reason why lrs was regarded as not suitable for EFM/Vs enumeration.

However, in 2017 Avis et al. presented an improved and parallel implementation of lrs [21]—the mplrs [1] algorithm. It uses message passing interface (MPI), a well-known interface for parallel computing architectures, together with an improved load balancing strategy to utilize up to 2000 cores in a cluster at once. The load balancing strategy divides processes into three categories: a *master*, a *consumer* and multiple *workers*. The *master* process handles the input data and runs in a main loop that consists of distributing sub-problems to the *workers* and receiving unfinished sub-problems from them. The *master* process starts by choosing an initial *worker* which it sends the initial sub-problem to. This *worker* then sends unfinished sub-problems back to the *master* as soon as its *budget* is exhausted or the problem is solved. The *budget* defines the maximum number of nodes that can be visited by a *worker*. It is dynamically calculated by mplrs depending on the number of available threads and the current size of the sub-problem list. As soon as the initial *worker* sends back unfinished sub-problems, the *master* starts distributing these sub-problems to all *workers* available. Each *worker* receives one sub-problem and only solves this assigned task. Unsolved sub-problems are sent back to the *master* and solved ones are sent to the *consumer* process which collects all results received from the *workers* and builds the output stream [1].

Duplicates are avoided as only solutions with a lexicographically minimum basis are printed. However, uniqueness can only be guaranteed when the polyhedron is a pointed cone with the origin as the only vertex $\varvec{\varepsilon }_0$. For unbounded polyhedra duplicates can occur during facet enumeration as there are multiple vertices. To guarantee unique solutions it would be necessary to keep a record of all solutions in memory in order to find the *true* minimum basis. Since this would increase memory requirements and negatively impact performance it was not included in the current version of mplrs. Hence, during EFV enumeration duplicates can occur [21].

Regarding lrs’s load balancing strategy, it becomes clear that *real* parallelization requires at least 4 processes, since two processes are always occupied by *master* and *consumer*. This way of balancing the load gives three main advantages: (i) *workers* can solve *their* assigned sub-problems independently, (ii) the required amount of RAM is distributed equally over a cluster and (iii) it’s possible to stop and later continue calculations from the last calculated result at any time [1].

These properties make mplrs [1] highly scalable and an ideal algorithm for HPC. In the context of metabolic networks and EFM/V analysis, mplrs is a completely new approach and the first that can truly utilize state of the art high-performance systems. Thus, EFM analysis is no longer bound to a single server, does not require an incredible amount of RAM, but only depends on the number of available CPU cores in a shared memory cluster.

## EFMlrs

The main purpose of EFMlrs is the pre- and post-processing of metabolic models for enumeration of EFM/Vs on HPC clusters via mplrs [1]. In addition, EFMlrs also supports EFM enumeration via efmtool [12].

EFMlrs is an open-source program, implemented in python 3 under the GPLv3 license, supported on Linux and macOS. Common python libraries (*COBRApy*, *NumPy*, *SymPy* and *Pandas*) are used for parsing sbml models, numerical computation and data processing. EFMlrs is part of the python package index and can therefore be easily installed via pip. The complete python code, further documentation as well as a tutorial are available on GitHub (http://github.com/BeeAnka/EFMlrs). Additionally, detailed documentation about the structure of the code and the functions used can be accessed on https://efmlrs.readthedocs.io.

**Fig. 3:**
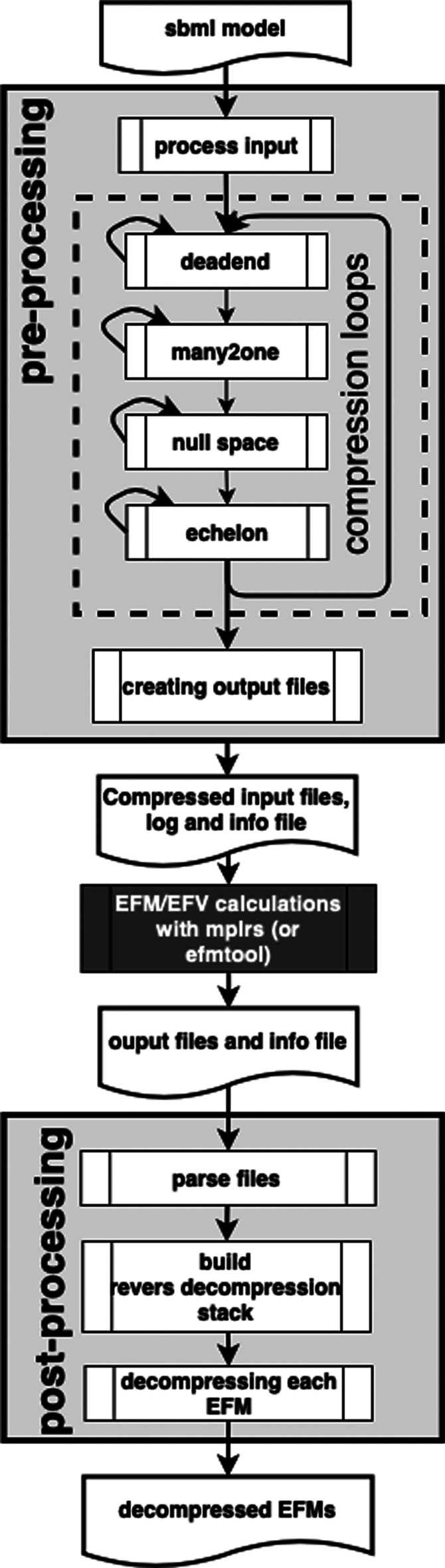
Overview of the EFMlrs workflow. It consists of three main stages: pre-processing, computation of EFM/Vs, and post-processing. The computations are intentionally not directly included in the program as mplrs [1] is meant to be executed on a HPC cluster, whereas pre- and post-processing, as well as computations with efmtool [12], can be done on a single machine

EFMlrs comes together with a designated workflow that consists of three stages: pre-processing metabolic models including transformation and loss-free compressions of the stoichiometric matrix, computations of EFM/Vs in the compressed system, and post-processing which includes decompression of EFM/Vs. An overview of the complete workflow is given in Fig. 3.

The computations are intentionally not directly included in the program as mplrs [1] is meant to be executed on a HPC cluster or multiple servers with shared memory. Although efmtool [12] can be executed on a desktop machine too, due to its high memory requirements it is recommended to use a server. Pre- and post-processing on the other hand can be executed on a desktop machine and represent core elements of EFMlrs. Besides, this setup gives the users more flexibility as switching between computing platforms is already taken into consideration, especially since computing time on HPC systems is often limited.

### Compressions

The compression algorithms used by EFMlrs are already known in the metabolic modeling community, have been discussed e.g. by Gagneur et al. [22] and have been implemented in e.g. efmtool [12]. Additional file 1: Table S1 compares the compression results of EFMlrs and efmtool. Since the compressions of both tools are based on the same algorithms, their results are very similar as well. However, for 3 out of 4 models (Table 1) used in this paper, EFMlrs could achieve a *stronger* compression and efmtool was not able to further compress any models compressed by EFMlrs. Only for the *EColiCore2* [29] model the compression results of both tools were equivalent. A compression comparison the other way round - first compressions by efmtool and then by EFMlrs could not be done, since efmtool does not output the compressed files needed for this.

**Table 1:** Overview of metabolic models and general polyhedra used for performance tests and comparisons of efmtool [12] and mplrs [1]. For metabolic models the number of rows corresponds to the number of metabolites and the number of columns to the number of reactions with numbers in brackets referring to the number of reversible reactions. For the metabolic models the dimensions of the original uncompressed model and a comparison between the compressions of efmtool and EFMlrs are given. For the polytopes, the matrix dimensions of the original uncompressed (uncmp.) polytope P, the transformed uncompressed and via efmtool compressed model in the flux cone FC are shown. All polytopes were obtained from the lrs homepage [27]. comp., compression; Deg., degree of degeneracy

Model	uncmp. *P*		uncmp. *FC*		efmtool comp.		EFMIrs comp.		EFMs	Deg.
	Columns	Rows	Columns	Rows	Columns	Rows	Columns	Rows		
EColiCentral [28]			71 (15)	53	44 (11)	26	44 (11)	21	429,276	High
EColiCore2 [29]			82 (22)	54	58 (18)	30	58 (18)	30	34,896,477	High
*i*PS189 [30]			277 (21)	271	67 (13)	42	63 (13)	35	3,252,686	High
JCVI-syn3A [31]			316 (8)	286	100 (7)	50	100 (7)	48	?	High
cp6 [27]	16 (15)	368	384 (15)	368	384 (15)	368			32	High
bv7 [27]	57 (56)	69	106 (56)	69	49 (0)	12			5,040	High
mit71 [27]	61 (60)	71	132 (60)	71	132 (60)	71			3,149,579	Moderate
fq48-19 [27]	19 (18)	48	67 (18)	48	48 (0)	29			119,184	Moderate
mit [27]	9 (8)	729	738 (8)	729	729 (0)	720			4,862	Moderate
perm10 [27]	11 (10)	1023	1033 (10)	1023	1033 (10)	1023			3628800	Simple

We attribute the slightly different compression results to the different order and number of iteration steps found in the respective implementations of the compression algorithms. However, at this moment the exact reason is unknown and subject of future investigations. It should be noted that the *stronger* compressions of EFMlrs together with the implementation in Python and the usage of *SymPy* lead to longer compression times compared to efmtool [12] which is implemented in Java - a statically typed and compiled and therefore faster programming language compared to Python. However, since in contrast to efmtool the compressions of EFMlrs are not directly coupled with the following calculations and the compressions have to be done only once, the overall time loss is not a big factor when calculating large models. Further details on EFMlrs workflow, the compression algorithms used and a visual comparison between an uncompressed and a compressed metabolic network (see Additional file 1: Figure S1) are provided in the Additional file 1.

## Results and discussion

In the following, the different requirements and performances of mplrs [1] and efmtool [12] are evaluated and compared. The main aspects of these analyses are run time and memory requirements, since these two factors are the main obstacles preventing further widespread use and application of EFM analysis. Also, the scaling behavior of the different parallelization techniques is examined and compared in more detail, as well as the performance of the two algorithms when computing models with different degrees of degeneracy. Furthermore, we investigated which formulation is most suitable for computations with mplrs and show that a “minimal” cell is maybe not so minimal at all.

All metabolic models and polytopes used in this work are summarized in Table 1. The wall time and the RAM requirements were obtained using the time command. Calculations were partly performed on servers provided by acib [32] and partly on the Vienna Scientific Cluster (VSC) [33].

### Comparison of run times, memory requirements and scaling behavior of efmtool [12] and mplrs [1]

**Fig. 4:**
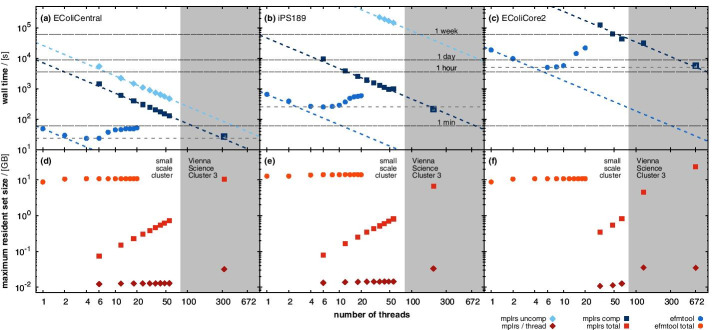
Performance comparison of efmtool [12] (circles) and mplrs [1] (full squares) enumerating EFMs in the flux cone (9) of the three compressed metabolic models: EColiCentral [28] (**a**, **d**), EColiCore2 [29] (**b**, **e**) and *i*PS189 [30] (**c**, **f**). Top panels compare run time as function of thread number. Additionally, we checked mplrs’s run time behavior for the uncompressed models (triangles). Dotted lines indicate efmtool’s minimum wall time, and a power-law fit to mplrs’s wall time, respectively. The intersections of the dotted lines mark (predicted) points where mplrs is as fast as the fastest efmtool run. We validated these points by running mplrs with the predicted number of threads (open squares). Note, open squares were not used in the power-law fit. The 4 thin gray lines indicate 1 min, 1 h, 1 day and 1 week—as stated in the upper middle plot. Bottom panels compare maximum resident set size during EFM enumeration as function of thread number. For mplrs, we also plotted the maximum resident set size per thread (open squares). Two different clusters were used for this analysis as indicated by the different background shadings

We enumerated all EFMs in the flux cone (9) of the metabolic models EColiCentral [28], EColiCore2 [29], and *i*PS189 [30] with efmtool and mplrs. Figure 4 illustrates a run time comparison between both tools as function of the number of available threads. In single-thread mode efmtool enumerates EFMs many times faster than lrs [21]. Only when using several hundred threads does mplrs become as fast as efmtool, see Fig. 4.

efmtool [12] scales rather poorly with the number of threads. On average already with two threads, efmtool loses 12.5% of the maximally achievable parallelization gain. Apparently independent of the enumeration problem, efmtool works fastest with approximately six threads, see Fig. 4. However, with six threads efmtool utilizes only 53.3% of the ideal parallelization gain. More than six threads obstruct each other and any parallelization gains are quickly lost if the number of threads is increased further. This may not be a problem for small models, which can be analyzed on desktop computers, but it essentially excludes the use of highly parallelized HPC infrastructures.

In contrast, mplrs [1], although rather slow when using only a few threads, is almost ideally parallelizable and – when run with a few hundred threads—outperforms efmtool [12], see Fig. 4. Lossless network compression strongly improves mplrs’s performance.

efmtool’s run time advantage comes at the price of enormous memory consumption. Even the smallest model with $\approx {0.5}\,\times \,10^{6}$ EFMs required already 10 GB, see Fig. 4. Shared memory demand rises further for the two larger models reaching 25 GB for the enumeration of $\approx {35}\,\times \,10^{6}$ EFMs in EColiCore2 [29]. The large memory consumption essentially limits the scalability of efmtool [12] and reflects the fact that during the iteration phase the DDM constructs many intermediate EFMs that are not elements of the final polyhedron, see the red vertex in Fig. 1. However, in all cases, the maximum memory consumption was essentially independent of the number of available threads. This is a characteristic of the DDM, which requires storing all intermediate results. Hence, more threads do not give an advantage concerning to the amount or distribution of required shared memory.

This is in strong contrast to mplrs’s performance, which consumes a constant, machine-dependent but very small amount of memory (several ten MB) per thread. Thus, total memory consumption scales linearly with the number of threads and is—in contrast to the efmtool [12]—no longer limiting.

**Fig. 5:**
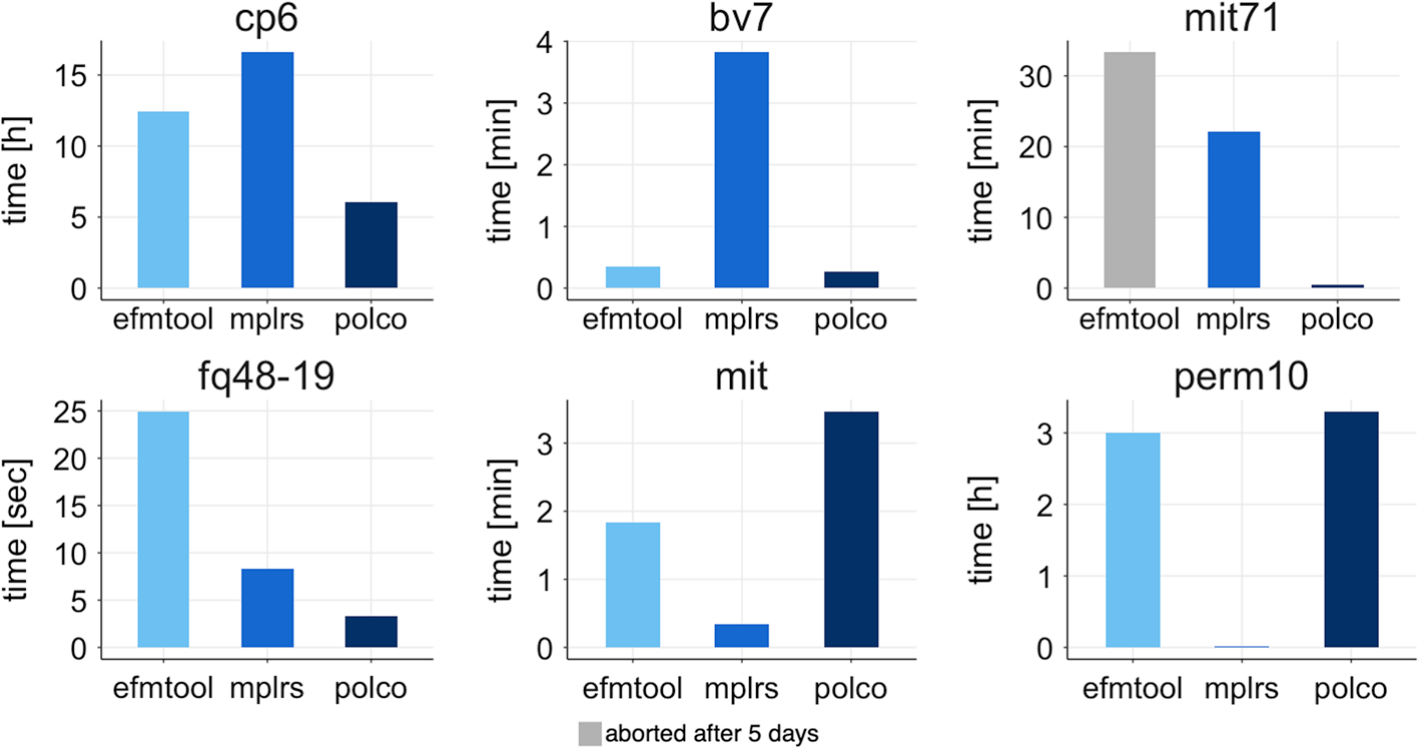
Comparison of the run times for six general polyhedra with different grades of degeneracy of mplrs [1] and two representatives of the DDM—efmtool and polco [12]. The y-axis shows the measured wall time in units suitable for the respective calculations, varying from seconds to hours. The x-axis shows the results of the three tools. 20 threads were used for all calculations. For calculations with polco and mplrs, the models were taken in the *H-representation* as provided on the lrs homepage. Only for use with efmtool, the input matrices first needed to be transformed into a flux cone (9) as described in the method section. To make the comparison as fair as possible no compressions–neither through EFMlrs nor through internal compression methods of polco or efmtool, were applied and no output files were written. The models are sorted in descending order according to their degree of degeneracy. From *cp6* and *bv7* being highly degenerate to *perm10* a simple 9-dimensional polytope. The DDM-based tools were faster for the high to moderate degenerate models, while mplrs outperformed both efmtool and polco on the simple models. For the calculations of the *mit71* polco needed 33 s and mplrs 22 min. Unfortunately calculations with efmtool could not be finished and were aborted after 5 days. In the plot this is indicated by a gray column. All in all, our results confirm the widespread assumption that the DDM is faster for degenerate polyhedra, whereas mplrs was faster for the simple polyhedra. This can be seen particularly well in the perm10 model for which mplrs required less than a minute while the DDM based methods needed a bit more than 3 h

Next, we wanted to compare the performances of the DDM and mplrs [1] with a fixed number of threads in enumerating extreme rays and extreme vectors in six general polyhedra of various degeneracy, see Fig. 5. Since efmtool [12] is specialized on metabolic models, we also included polco [12], another but more general implementation of the DDM, in this comparison. All of the models used are freely available for download on the lrs homepage [27]. For computations with mplrs and polco, the polyhedra were taken as provided in the *H-representation*. For use with efmtool, the input matrices needed to be transformed into a flux cone (9) as described in the method section. To ensure the fairest possible comparison no compressions—neither through EFMlrs nor through internal compression methods of polco or efmtool—were applied and no output was written.

Figure 5 shows the results of this comparison. For highly to medium degenerate polytopes polco [12] and efmtool [12] were faster, with the exception being *mit71*, a moderately degenerate polytope. Within five days efmtool was unable to finish enumeration, while polco needed less than a minute and mplrs [1] less than 30 min. For the two simple polytopes (*mit* and *perm10*) mplrs was by far the fastest. The difference becomes most evident for the *perm10* model. Here the DDM based tools needed a bit more than 3 h, while mplrs was able to solve it in less than a minute. Additionally, looking at the average amount of RAM needed for calculating one model, it shows that the fast performance of DDM-based methods is *payed* in memory. On average, polco needed 153.5 GB, efmtool 116.2 GB and mplrs 0.2 GB of RAM per model. Our results are well in line with conventional wisdom that mplrs is faster for non-degenerate models, whereas DDM-based methods, like efmtool or polco, perform better in highly degenerate cases [19], but require a huge amount of memory.

### Performance of mplrs in different geometric shapes

mplrs [1] offers the possibility to formulate the EFM enumeration problem in different geometric objects, i.e. as in (5) as polyhedron *P*, as in (7) as polyhedral cone *PC*, or as in (9) as flux cone *FC*. Thus, we tested if this flexibility allows us to accelerate mplrs’s performance.

We used EColiCore2 [29] subject to all combinations of constraints listed in Table 2 and computed all EFM/Vs in the different geometric formulations (flux cone, (9), polyhedral cone (7), and general polyhedron (5)) with mplrs [1]. Prior to the calculations, all uptake reactions including thereby orphaned metabolites for carbon uptake were removed with the exception of glucose. Table 3 lists the dimensions of the input matrices for these different scenarios. We observe that both run time and output size of mplrs heavily depend on the problem formulation.

**Table 2:** Bounds applied to the reactions of EColiCore2 [29]

ID	Bound [mmol/gh]	Description
R_GlcUp	$\le 10$	Glucose uptake
R_O2Up	$\le 5$	Oxygen uptake
R_ATPM	$\ge 3.15$	Maintenance demand

**Table 3:** Dimensions of the input matrices for the different scenarios of EColiCore2 [29] with various reaction bounds (see Table 2) applied. All matrices are compressed and redundant rows are removed. Compressions were applied through EFMlrs and redundant rows were removed using the lrs redund function [1]

Bound(s)	Geometry	Rows × columns
ATPM	FC, PC	107 $\times$ 82
	P	106 $\times$ 81
GlcUP O2Up	FC, PC	107 $\times$ 82
	P	107 $\times$ 81
ATPM,GlcUp & ATPM,O2Up	FC, PC	107 $\times$ 82
	P	107 $\times$ 81
GlcUP,O2Up & ATPM,GlcUp, O2Up	FC	109 $\times$ 83
	PC	108 $\times$ 82
	P	108 $\times$ 81

**Fig. 6:**
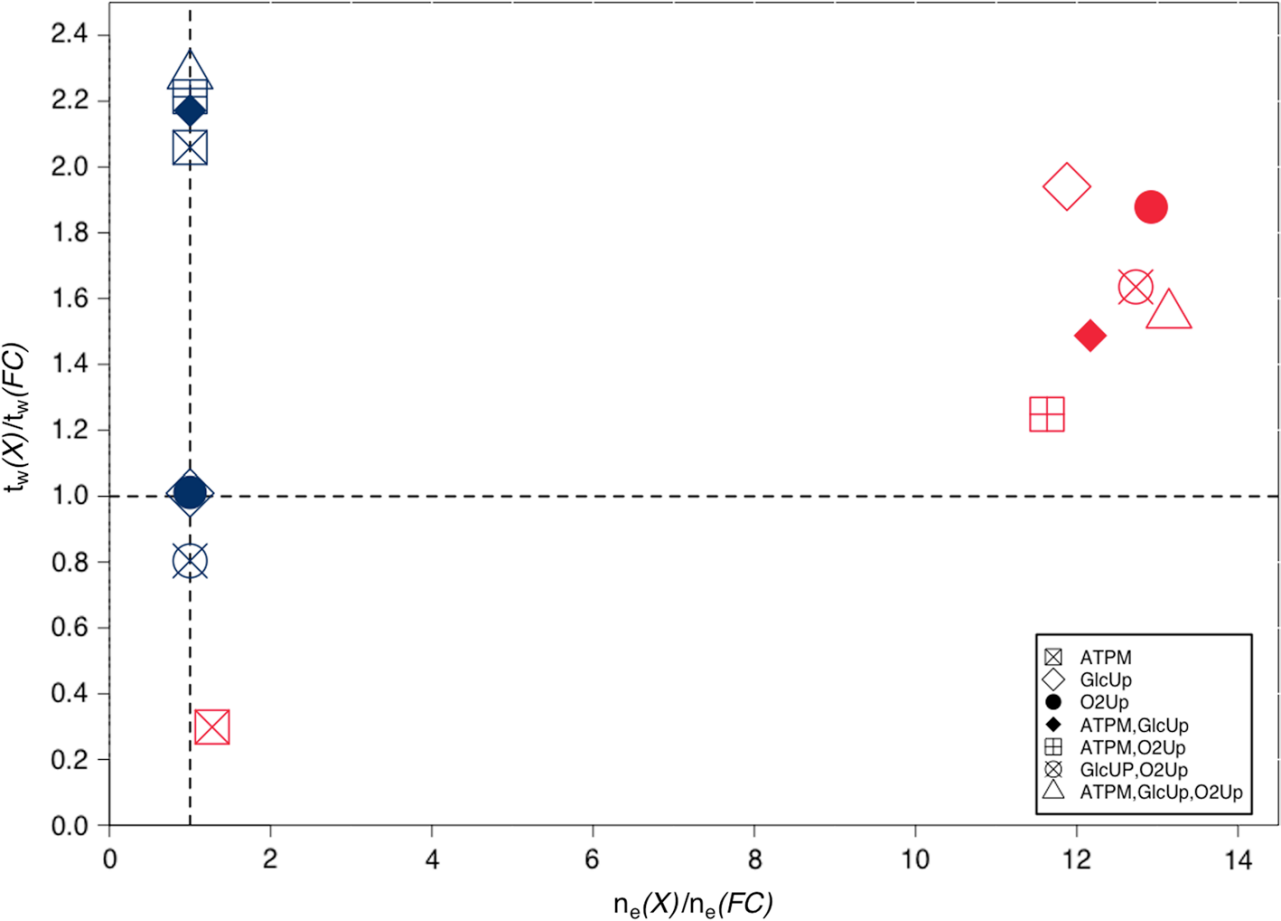
Visualization of the ratios between multiple possible occurrences of EFVs found in a general polyhedron *P* (5) and the set of unique EFVs found in the cone shapes (*FC* (9), *PC* (7)) of EColiCore2 [29] with different reaction bounds applied (Table 2). The x-axis shows the ratio between the number of EFVs found in the cone shapes and the number of EFVs found in the polyhedron. The y-axis shows the factor between the run times of a flux cone *FC* and a general polyhedron *P*, respectively a polyhedral cone *PC*. The results of *P* and *PC* are arranged on the y-axis according to their factor. Along the dashed vertical line at *(1, 0)* the results from the polyhedral cone *PC* (blue) are located on the x-axis and according to their uniqueness accounted for as *1*. The results from *P* (red) are arranged along the x-axis according to their multiples in comparison to the results of the cones. Each scenario was given a specific symbol as shown in the boxed legend

The smallest output size is achieved if the enumeration problem is formulated as a (flux or polyhedral) cone (see Additional file 1: Figure S2b). If, however, the problem is formulated as a general polyhedron still all EFM/Vs get enumerated but some are listed multiple times [27], which increases the output size. In our examples, every EFM/Vs gets enumerated 10.8 times on average. Figure 6 shows the ratios between multiple possible occurrences of EFM/Vs found in a general polyhedron and the set of unique EFM/Vs found in the cone shapes. Typically the larger output size of the general polyhedron increases the run time on average by a factor of 1.5 compared to the flux cone and by 1.2 compared to the PC. Yet, we found one case (ATPM) where the enumeration of EFM/Vs in a general polytope took 30% of time used in the flux cone formulation although the output size increased by 30%. Similarly, EFM/V enumeration in a polyhedral cone is on average 1.6 times slower than in the corresponding flux cone. Again we found one counterexample—the model with bounds on the reactions GlcUp and O2Up. Here the polyhedral cone was almost 25% faster compared to the flux cone. Overall, we concluded that it is generally better to use the flux cone formulation. A comparison of the run times and the different amounts of results found in the cone shapes versus the general polyhedron is provided in the Additional file 1: Figure S2.

### A not so minimal cell ...

*JCVI-syn3A* is a synthetic, minimal cell with a 543 kbp genome and 493 genes [31]. Its metabolic reconstruction contains 304 metabolites, 338 reactions in total including 244 non-pseudo reactions and 155 genes. Prior to enumerating EFMs, we made the model consistent. That is, we removed all dead-end metabolites and reactions that were unable to carry non-zero steady-state fluxes under any circumstances. Additionally, we redefined reversible reactions to be irreversible, if their fluxes never changed directions, see Table 1. Although *JCVI-syn3A* [31] is a reconstruction of a minimal cell it was not possible to enumerate all EFMs in the flux cone of this model via efmtool [12] on our computing infrastructure^1^.

To investigate the explosion of the number of EFMs in *JCVI-syn3A* [31] in more detail, we reduced *JCVI-syn3A* [31] as much as possible. For this purpose, we computed a minimal medium using the *COBRApy* function minimal_medium() that supports a maximum growth rate of 0.342. In total 22 uptake reactions were not required in this minimal medium and thus removed from the model. This model is referred to as $sm_0$. Besides, we created a series of 21 models $sm_i$ where each model $sm_i$ contained one additional uptake reaction (of the set of non-minimal uptakes) compared to its predecessor model $sm_{i-1}$ (models are available at https://github.com/BeeAnka/EFMlrs). Hence, we got 22 sub-models. The smallest ($sm_0$) of which modeled growth on minimal medium, the largest ($sm_{21}$) misses one uptake reaction compared to the original model *JCVI-syn3A* [31]. Afterwards we enumerated EFMs in the flux cone (9) of these models with efmtool [12] and mplrs [1] starting with the smallest sub-model $sm_0$. In this sub-model ($sm_0$) we found 768,990 EFMs and calculations took approximately 2 min (efmtool 6 threads, mplrs 48 threads). With efmtool we were able to enumerate EFMs in all sub-models up to $sm_7$. With mplrs we completely enumerated all sub-models up to $sm_{15}$. It contained 12,051,382,513 EFMs and enumerations took almost 5 days with 960 parallel threads on the VSC 4. Table 4 provides an overview of (selected) sub-models and associated milestones.

**Table 4:** Overview of the most important sub-models of *JCVI-syn3A* [31] and associated milestones

Name	Reactions total	Non-essential uptake reactions	EFMs	**Milestone**
*sm00*	253	0	768,990	Smallest sub-model
*sm04*	263	4	6,595,338	Sub-model used for yield analysis
*sm07*	272	7	52,761,066	Last calculations possible with efmtool
*sm08*	275	8	105,521,898	Last model with further process-able output size
*sm15*	296	15	12,051,382,513	Last model calculated yet
*full model*	316	22	*unknown*	Includes all non-essential exchange reactions

**Fig. 7:**
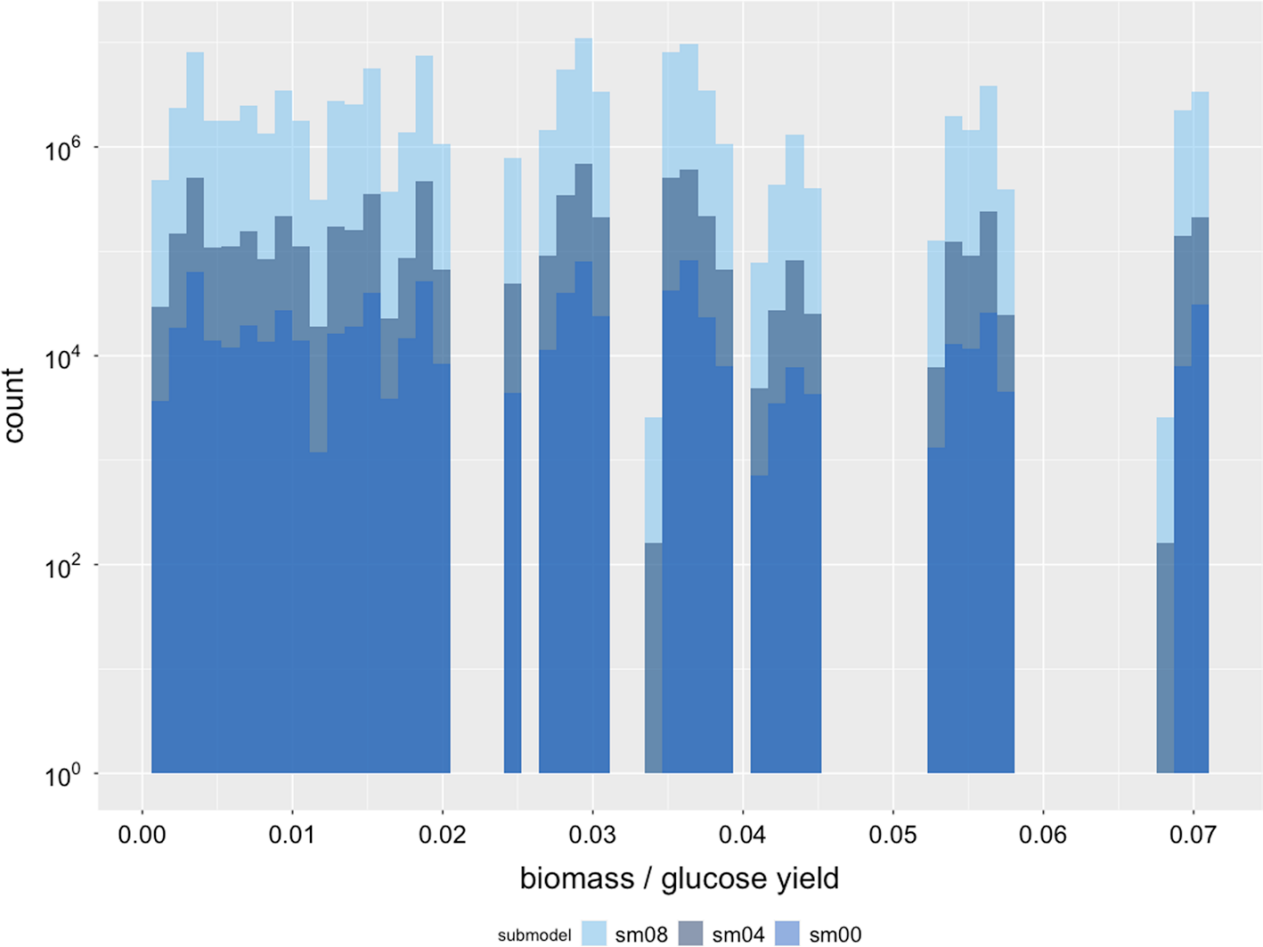
Histogram of the biomass / glucose yield for the sub-models $sm_0$, $sm_4$ and $sm_8$ of *JCVI-syn3A* [31]. The x-axis shows the yield and the y-axis the count in logarithmic scale. The sub-models are plotted in different colors on top of each other with the smallest sub-model $sm_0$ in dark blue in the top, sub-model $sm_4$ in gray in the middle and the largest sub-model $sm_8$ in light blue in the bottom layer. The factor between $sm_4$ and $sm_8$ is exactly 16 over the complete distribution, the factor between $sm_0$ and $sm_4$ is 8.6 and 138.3 between $sm_0$ and $sm_8$. This shows that the bigger sub-models completely include all previous smaller sub-models and that additional carbon sources do not lead to new metabolic pathways, but rather integrate into and reinforce existing structures

**Fig. 8:**
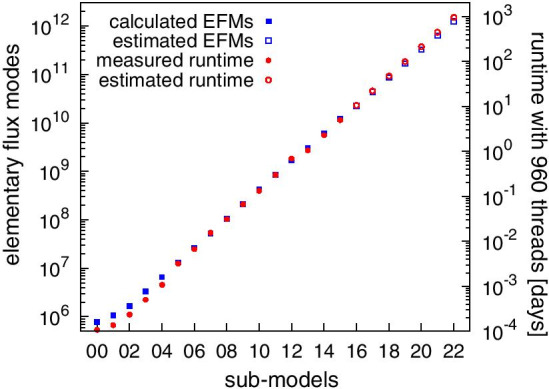
Visualization of the scaling behavior of the sub-models of *JCVI-syn3A* [31]. The plot shows the correlation between the included number of non-essential uptake reactions, the number of resulting EFMs, and the run times. The sub-models are plotted on the x-axis and referred to by their number e.g. 00 refers to $sm_{00}$. Y-axis 1 shows the number of calculated/predicted EFMs, y-axis 2 the required/predicted run time in days using 960 threads on the VSC. Both axes are in logarithmic scale. Full symbols (dots and squares) refer to measured run times and computed EFMs (sub-models $sm_{00}$ to $sm_{15}$). The corresponding equations for the non-logarithmic representation of the calculated results are $y1=264629e^{0.6678x}$ with $R^2=0.9997$ and $y2=3E^{-5}e^{0.7508x}$ with $R^2=0.9986$. Empty symbols refer to estimated data (sub-models $sm_{16}$ to $sm_{22}$) with the assumption that the slopes for both curves remain the same over all sub-models. Sub-model $sm_{00}$ refers to the *smallest* sub-model that includes none of the 22 non-essential uptake reactions and $sm_{22}$ to the *full* model that includes all uptake reactions

For all enumerated sub-models we confirmed (i) that mplrs [1] and efmtool [12] returned an identical set of EFMs, and (ii) that the set of EFMs in the preceding models $sm_{i-1}$ are completely contained in the set of EFMs in the successive model $sm_i$. The latter is illustrated in Fig. 7, where we plotted the (discontinuous) distribution of the biomass yield on glucose over the set of EFMs in the sub-models $sm_0$, $sm_4$, and $sm_8$. Comparing the yield distributions of the individual sub-models shows that their ratios are relatively constant. In fact, the factor between the yield distributions of $sm_4$ and $sm_8$ is exactly 16 over the complete distribution and although the other two factors (8.6 between $sm_0$ and $sm_4$, and 138.3 between $sm_0$ and $sm_8$) have a slightly greater variance, they remain relatively constant as well. We further observe that additional growth sources rarely narrow the gaps in the biomass yield distribution (see e.g. at around 0.033 or 0.067), but rather increase the count of already existing ones. This indicates that additional carbon sources mainly couple into the existing pathway structure, rather than contributing some completely new functionality. Additional support for this conclusion comes from the observation that the number of EFMs approximately doubles with every additional uptake reaction, see Fig. 8. We validated this trend for the first 16 of 22 sub-models. If this tendency continues, *JCVI-syn3A* [31] will have more than one trillion EFMs. At the VSC, their complete enumeration would take more than 2.5 years with 960 threads and the compressed output file would be about $33 \times 10^6$ GB in size.

**Fig. 9:**
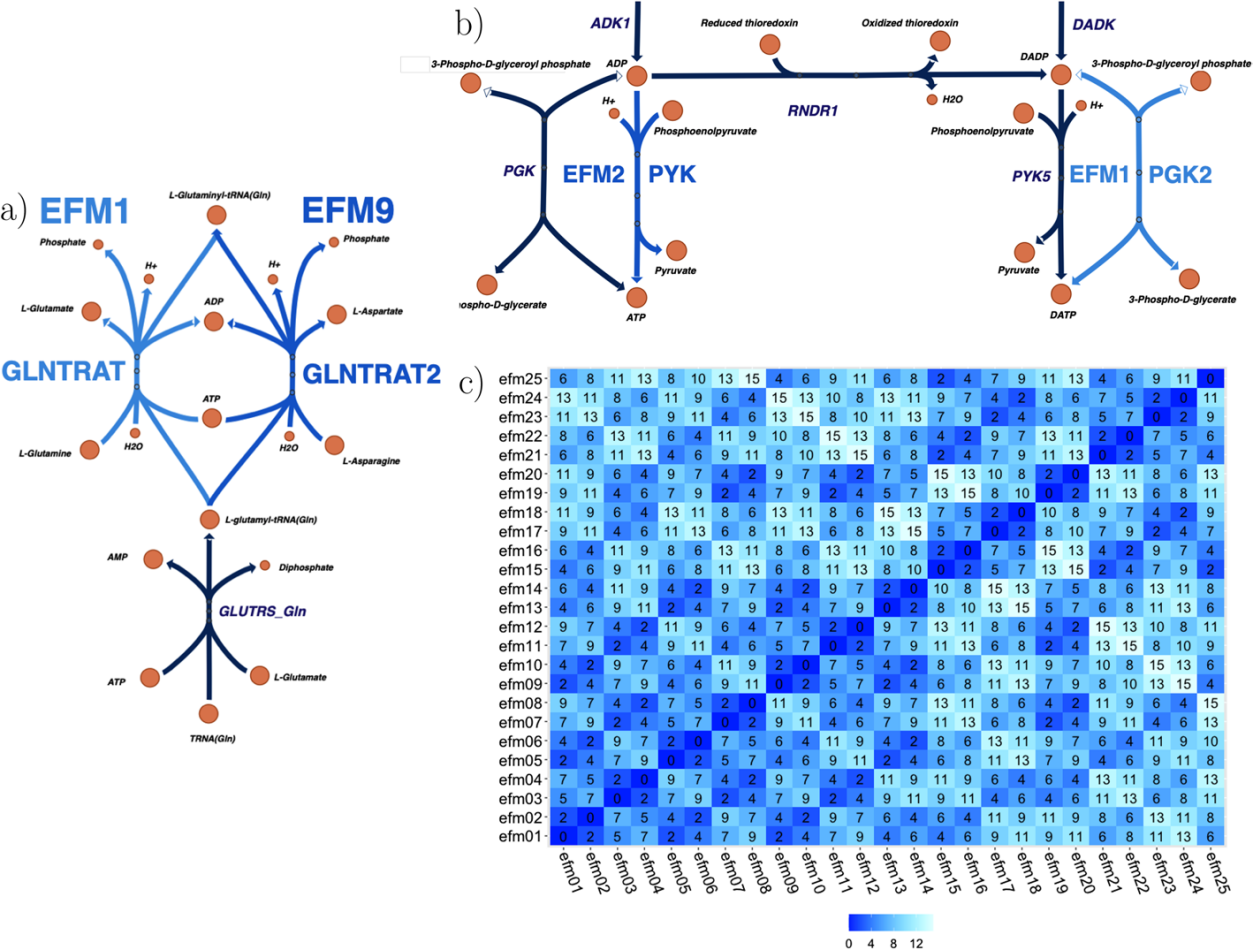
The three Figures show different visualizations of EFMs that belong to the 15,360 identified highest yield EFMs of the sub-model $sm_8$ of *JCVI-syn3A* [31]. Each EFM consists of 275 reactions in total. **a** and **b** show excerpts of the pathways of EFM 1 and EFM 9 respectively EFM 2 where the respective EFMs only differ in two reactions from each other. Shared reactions are in dark blue whereas individual reactions are in the same color as the EFMs’ name, e.g. in **a** the reaction *GLUTRS_Gln* is the shared reaction, reaction *GLNTRAT* belongs to EFM 1 and *GLNTRAT2* belongs to EFM 9. **c** is a heat map of the top 25 highest yield EFMs, showing the number of reactions in which the EFMs differ from each other. Since EFM 1 differs from EFM 2, respectively EFM 9, in two reactions, consequently EFM 2 and EFM 9 differ in 4 reactions, indicated in the heat map by the number as well as a darker shade of blue. The darker the shade of blue, the greater the similarity between 2 EFMs

However, how can it be that in a *minimal* genome cell this astonishing number of EFMs can be found? To clarify this question, we made further efforts to analyze the biggest set of EFMs still process-able ($sm_8$). In a first step, we could identify 15,360 EFMs with the highest yield of 0.0705507. By analyzing and comparing the fluxes of these EFMs it became clear that although they are not identical, they are very similar. Each of the 15,360 EFMs differs only in two reactions from at least 5 other EFMs. On average an EFM differs from 6.2 other EFMs in exactly 2 reactions. This means that the set of EFMs, although still unique, at least partly consists of extremely similar EFMs. Figures 9a, b show examples of such similar EFMs. Each Figure displays a certain excerpt of the metabolic network of *JCVI-syn3A* [31] representing two EFMs that share 273 reaction fluxes and differ only in the remaining two. Figure 9c is a heat map of the top 25 highest yield EFMs, visualizing the differences between them. The EFMs from Fig. 9a, b are included in this map as well. The heat map shows that EFM 1 differs from EFM 2, respectively EFM 9 in only two reactions, consequently EFM 2 and 9 differ in only 4 reactions from each other. Even though only excerpts from the overall results could be analyzed, it becomes very clear how similar these EFMs are. Hence, it can be assumed that this pattern is present in the rest of the set of EFMs as well. To explore these results in more detail, further sub-set analysis e.g. by using ecmtool [34] or ProCEMs-enumeration [16], would be required. However, our analysis at least in part explains this incredible number of EFMs found in the minimal cell model *JCVI-syn3A* [31].

## Conclusion

efmtool [12], respectively the DDM, remains essential for complete enumeration and analysis of EFMs in small-scale metabolic networks. Yet, it is memory intensive, lacks scalability and its application is therefore limited. In contrast, mplrs [1] allows for scalable, massively parallel enumeration of EFMs, which is no longer limited by (expensive) RAM restrictions but (cheap) computing power. Given the increasing power and availability of HPC clusters and cloud-services, mplrs opens new possibilities to overcome current limitations. To facilitate such a transformation EFMlrs provides a Python framework to harvest these promises for an unbiased characterization of metabolic networks. Additionally, the concept to generalize EFMs from flux cones to flux polyhedra, already introduced by Urbanczik over a decade ago [5], is incorporated in EFMlrs as well. In the future, mplrs could probably also be used in conjunction with other programs, such as ecmtool [34], as an alternative to the DDM and make these tools even more efficient.

EFMlrs is the first program that gives users the ability to enumerate EFM/Vs in metabolic models on HPC clusters via mplrs [1]. It can be used as a stand-alone program but also seamlessly integrates in existing workflows. In particular, EFMlrs adds (i) the possibility to calculate EFVs to efmtool and *COBRApy* and (ii) opens the doors to HPC systems for EFM/V analysis via mplrs.

## Supplementary Information

**Additional file 1:** More detailed information on EFMlrs compression algorithms incl. detailed description, pseudo-code snippets and comparison of different geometric formulations.

## Data Availability

EFMlrs is an open-source program that comes together with a designated workflow. It’s implemented in Python 3, listed in the Python Package Index, and can be easily installed via pip. The complete source code, documentation and a tutorial are available on https://github.com/BeeAnka/EFMlrs.

## Footnotes

1 CPU model: Intel(R) Xeon(R) Silver 4114 CPU @ 2.20GHz, sockets: 2, CPU cores: 10, total threads: 40, 825 GB RAM
