# Supplementary material for: EFMlrs: a Python package for elementary flux mode enumeration via lexicographic reverse search
Source: BMC Bioinformatics. 2021 Nov 10;22:547. doi: 10.1186/s12859-021-04417-9 (PMC8579665; doi:10.1186/s12859-021-04417-9)
Supplement: Supplementary file 1 — Additional file 1: More detailed information on EFMlrs compression algorithms incl. detailed description, pseudo-code snippets and comparison of different geometric formulations. [file 12859_2021_4417_MOESM1_ESM.pdf]

Supplementary material
`EFMlrs`
Compressions
The compression algorithms used by `EFMlrs` are already known in the metabolic modeling community, have been discussed e.g. by Gagneur et al. [22] and have been implemented in e.g. `efmtool` [12]. Table S1 compares the compression results of `EFMlrs` and `efmtool`. Since the compressions of both tools are based on the same algorithms, their results are very similar as well. However, for 3 out of 4 models (Table 1) used in this paper, `EFMlrs` could achieve a *stronger* compression and `efmtool` was not able to further compress any models compressed by `EFMlrs`. Only for the *EColiCore2* [29] model the compression results of both tools were equivalent. A compression comparison the other way round - first compressions by `efmtool` and then by `EFMlrs` could not be done, since `efmtool` does not output the compressed files needed for this.

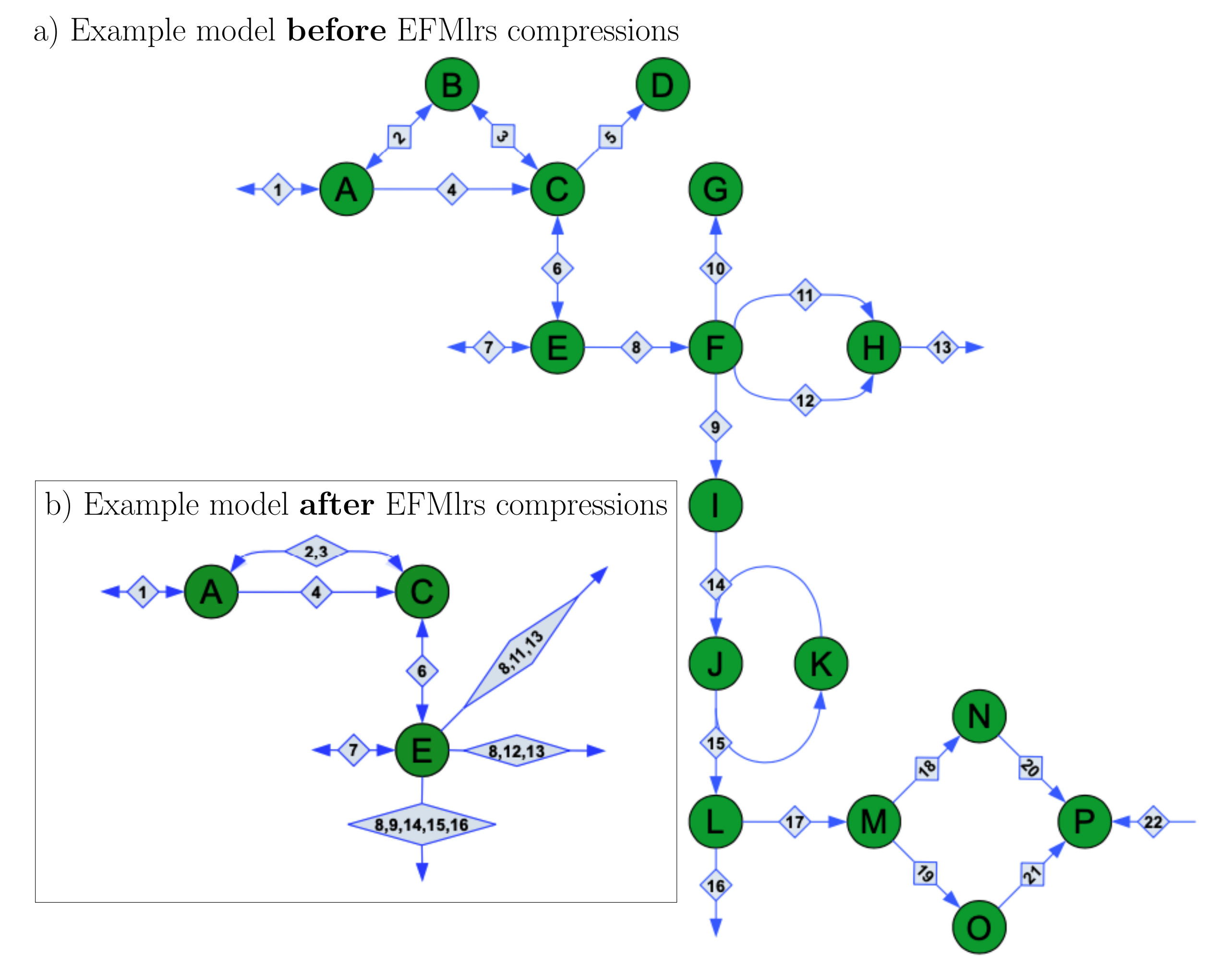

Figure S1 Example of a metabolic network. Figure S1 a shows the original network without any compressions. Figure S1 b shows the network after loss-free compressions were applied using `EFMlrs`. The original (uncompressed) model consists of 22 reactions (2 reversible) and 16 metabolites. The compressed model accommodates 8 reactions (1 reversible) and 3 metabolites.

We attribute the slightly different compression results to the different order and amount of iteration steps found in the respective implementations of the compression algorithms. However, at this moment the exact reason is unknown and subject of future investigations. It should be noted that the *stronger* compressions of `EFMlrs` together with the implementation in Python and the usage of *SymPy* lead to longer compression times compared to `efmtool` [12] which is implemented in Java - a statically typed and compiled and therefore faster programming language compared to Python. However, since in contrast to `efmtool` the compressions of `EFMlrs` are not directly coupled with the following calculations and the compressions have to be done only once, the overall time loss is not a big factor when calculating large models. For a detailed description of `EFMlrs` workflow and the compression algorithms see the supplementary material.

`EFMlrs` workflow
pre-processing
The `EFMlrs` workflow begins with parsing a sbml input file that contains the model of interest. Therefore, `EFMlrs` utilizes *COBRApy* functions for parsing and preparing metabolic models and thereby features model editing if necessary or desired e.g. excluding specific compartments or specifying if non-default reaction bounds should be taken into account, thus resulting in computing EFVs instead of EFMs.

Table S1 Compression comparison of `EFMlrs` versus `efmtool`. Values in table are reactions (reversible reactions) / metabolites.

	uncompressed model	`EFMlrs` compressions	`efmtool` compressions
EColiCentral [28]	71 (15) / 53	44 (11) / 21	44 (11) / 26
EColiCore2 [29]	82 (22) / 54	58 (18) / 30	58 (18) / 30
*i*PS189 [30]	277 (21) / 271	63 (13) / 35	67 (13) / 42
JCVI-syn3A [31]	316 (8) / 286	100 (7) / 48	100 (7) / 50

loss-free compressions of $\boldsymbol{N}$

Parsing the model is followed by the most important step during pre-processing – the loss-free compression of the stoichiometric matrix $\boldsymbol{N}$. Iteratively, four different loss-free compressions are applied on $\boldsymbol{N}$. All compression steps are executed one after another in a large outer loop, with each compression step itself being executed within a smaller loop. The inner loops of the single compression steps are executed until no more redundancies are found during this step. Only then the next compression step starts. After all compression steps have been run through, the large outer loop starts again until no redundancies can be found during any compression step. The complete sequence of pre-processing is illustrated in the top part of Figure 3. A pseudo-code snippet for each compression step is shown in the Algorithms 1, 2, 3 and 4.

The targets of the first compression step (Algorithm 1) are so-called dead-end metabolites. The metabolites D, G and P in Figure S1 a are examples for such metabolites. They can be identified by analyzing the rows of the stoichiometric matrix $\boldsymbol{N}$. Rows that contain only values with the same sign, indicate that the corresponding metabolites are only produced (positive values) respectively consumed (negative values). Therefore they can not be in a steady-state and consequently, they, as well as their contributing, irreversible reactions, can be removed from the network [12]. Thus the metabolites D, G and P together with their corresponding reactions can be removed from the metabolic network.

Algorithm 1 Pseudo-code snippet: *deadend*

function FINDS AND REMOVES REDUNDANCIES DUE TO DEADEND METABOLITES($\boldsymbol{N_{m,n}}$, $\boldsymbol{rev}$)
 ▷ finds and removes deadend metabolites and corresponding irreversible reactions from $\boldsymbol{N}$
 ▷ $\boldsymbol{N}$ is the stoichiometric matrix that denotes $\boldsymbol{m} \times \boldsymbol{n}$
 ▷ $\boldsymbol{rev}$ is a vector containing the reaction reversibilities
  for $\boldsymbol{m} \in \boldsymbol{N}$ **do**
   if all n $\in \boldsymbol{m} \geq 0$ **then**
    if all $\boldsymbol{rev}_{i \in \boldsymbol{n}_i > 0}$ = False **then**
     remove $\boldsymbol{m}$ from $\boldsymbol{N}$
    end if
   else if all n $\in \boldsymbol{m} \leq 0$ **then**
    if all $\boldsymbol{rev}_{i \in \boldsymbol{n}_i < 0}$ = False **then**
     remove $\boldsymbol{m}$ from $\boldsymbol{N}$
    end if
   end if
  end for
  for $\boldsymbol{n} \in \boldsymbol{N}$ **do**
   if $|\boldsymbol{n}| = 0$ **then**
    remove $\boldsymbol{n}$ from $\boldsymbol{N}$
    remove $\boldsymbol{rev}_n$ from $\boldsymbol{rev}$
   end if
  end for
  return $\Delta\boldsymbol{N}$, $\Delta\boldsymbol{rev}$
end function

The next compression step (Algorithm 2) is called *many2one* as during this step, reactions with unique fluxes are merged together. To further illustrate what unique fluxes mean, let's have a look at metabolite F in Figure S1 a. F is only produced by reaction $R8$ and afterward consumed by the reactions $9, 11, 12$ (note that $R10$ has already been removed during dead-end compression). So unique fluxes can easily be identified by analyzing the rows of $\boldsymbol{N}$. Thus the rows corresponding to uniquely produced (respectively consumed) metabolites have one positive entry and otherwise only negative entries, respectively one negative entry and otherwise only positive entries. Since we operate under steady-state conditions, $R8$ has to carry a non-zero flux, whenever the consuming reactions of F are active. Hence, it's possible to merge the sequential consuming (respectively producing) reactions and thereby further decrease the dimensions of the stoichiometric matrix $\boldsymbol{N}$ [22]. So, during a first iteration step of the *many2one* compression, we can lump the reactions $R8 + R11$, $R8 + R12$ and $R8 + R9$ together and remove the metabolite F. Note that this procedure is executed in loops and already merged reactions can be merged further with other reactions if during a next iteration further redundancies are found and the preconditions for the compressions are met. This applies to all compression steps.

Redundancies that only can be detected by analyzing the kernel K of the stoichiometric matrix $\boldsymbol{N}$ are the targets of the next compression step (Algorithm 3), the *nullspace* compression. If two rows in the kernel matrix K only differ from each other by a constant factor, also their fluxes only differ by this factor. If there is a flux through one of these reactions, there has to be a flux through the other, respectively if one reaction has no flux the other reaction is also passive. In Figure S1 a $R2$ and $R3$ are examples for such reactions that are called coupled reactions as they are co-regulated [12, 35]. Thus they are *forced*

Algorithm 2 Pseudo-code snippet: *many2one*

function FINDS REDUNDANCIES DUE TO UNIQUE FLUXES($\boldsymbol{N}_{\boldsymbol{m},\boldsymbol{n}}$, $\boldsymbol{rev}$)
▷ finds and merges reactions with unique fluxes in $\boldsymbol{N}$
▷ $\boldsymbol{N}$ is the stoichiometric matrix that denotes $\boldsymbol{m} \times \boldsymbol{n}$
▷ $\boldsymbol{rev}$ is a vector containing the reaction reversibilities
for $\boldsymbol{m} \in \boldsymbol{N}$ **do**
skip $\leftarrow$ False
for n $\in \boldsymbol{m}$ **do**
if n $\neq 0$ and $\boldsymbol{rev}_n = 1$ **then**
skip $\leftarrow$ True
end if
end for
if skip = False **then**
for n $\in \boldsymbol{m}$ **do**
if n ≥ 0 **then**
$\boldsymbol{a} \leftarrow \boldsymbol{a}$ append n
else
$\boldsymbol{b} \leftarrow \boldsymbol{b}$ append n
end if
end for
pos $\leftarrow |\boldsymbol{a}|$
neg $\leftarrow |\boldsymbol{b}|$
if (neg = 1 or pos = 1) and (neg + pos ≥ 2) **then**
if neg = 1 **then**
swap $\boldsymbol{a}$, $\boldsymbol{b}$
end if
for i $\in \boldsymbol{b}$ **do**
$\boldsymbol{n}_a \leftarrow \boldsymbol{n}_i \;/\; |i| + \boldsymbol{n}_a \;/\; |a|$
end for
remove $\boldsymbol{n}_a$ from $\boldsymbol{N}$
remove $\boldsymbol{rev}_a$ from $\boldsymbol{rev}$
for $\boldsymbol{m} \in \boldsymbol{N}$ **do**
if $|\boldsymbol{m}| = 0$ **then**
remove $\boldsymbol{m}$ from $\boldsymbol{N}$
end if
end for
end if
end if
end for
return $\Delta\boldsymbol{N}$, $\Delta\boldsymbol{rev}$
end function

to work together, they can also be lumped into one reaction by applying their coupling factor. In the example network (S1 a), we can merge $R2$ and $R3$ into one single reaction and thereby remove one reaction and the metabolite B from the network.

Algorithm 3 Pseudo-code snippet:*nullspace*

function FINDS AND REMOVES REDUNDANCIES DUE TO COUPLED REACTIONS($\boldsymbol{N_{m,n}}$, $\boldsymbol{rev}$)
  ▷ finds coupled reactions in $\boldsymbol{K}$ and merges them in $\boldsymbol{N}$
  ▷ $\boldsymbol{N}$ is the stoichiometric matrix that denotes $\boldsymbol{m} \times \boldsymbol{n}$
  ▷ $\boldsymbol{rev}$ is a vector containing the reaction reversibilities
  $\boldsymbol{K}_{i,j} \leftarrow$ kernel matrix of $\boldsymbol{N}$
  for $i1 \in \boldsymbol{K}$ **do**
    for $i2_{i+1} \in \boldsymbol{K}$ **do**
      factor $\leftarrow i1$ / $i2_{i+1}$
      if factor $\neq$ None **then**
        $\boldsymbol{n}_{i1} \leftarrow \boldsymbol{n}_{i1} + \boldsymbol{n}_{i2}$ / factor
        if $\boldsymbol{rev}_{i2}$ = False **then**
          $\boldsymbol{rev}_{i1} \leftarrow$ False
        end if
        remove $\boldsymbol{n}_{i2}$ from $\boldsymbol{N}$
        remove $\boldsymbol{rev}_{i2}$ from $\boldsymbol{rev}$
        remove $i2$ from $\boldsymbol{K}$
      end if
    end for
  end for
  for $\boldsymbol{m} \in \boldsymbol{N}$ **do**
    if $|\boldsymbol{m}| = 0$ **then**
      remove $\boldsymbol{m}$ from $\boldsymbol{N}$
    end if
  end for
  return $\Delta\boldsymbol{N}$, $\Delta\boldsymbol{rev}$
end function

The last compression step (Algorithm 4) is called *echelon* compression, as the redundancies it targets can be identified by analyzing the reduced row echelon form of the stoichiometric matrix $\boldsymbol{N}$. These redundancies are caused by metabolites that are in a so-called conservative relation with each other, meaning that there is a linear dependency between them [22]. Such linear dependencies show in the transposed reduced echelon form as a column that breaks the diagonal pattern of ones. In Figure S1 a the metabolites K and J are linearly dependent on each other and thereby build a conservative relation. K can only be produced if J is produced. However, J as well can only be produced while K is being produced. Therefore the reactions $R14$ and $R15$ are strongly linked thus any change in the concentration of the metabolite J leads to the same change in the concentration of the metabolite K. Hence, one of the two metabolite, in this case K, can be removed from the stoichiometric matrix $\boldsymbol{N}$ without losing any necessary information.
If no more redundancies can be found during any compression step, the flux cone (9) is reconfigured by splitting all reversible reactions into two – a forward and a backward reaction. Thereby the flux cone is transformed for calculations with `mplrs`. Afterwards, the compressed input files for `mplrs` and `efmtool`, as well as a log file and an info file, concerning the performed compressions and later needed for decompressions, are created. Now, the computations using either `mplrs` or `efmtool` can be started.

post-processing
When calculating EFM/Vs from a loss-free compressed network the number of resulting EFM/Vs is the same as if the calculations were done with the original network. However, the EFM/Vs are compressed and are still containing the merged reactions. Additionally, `mplrs`' output files also contain the previously split reversible reactions which need to be lumped together again. Thus, post-processing and decompression are needed to get the *full* set of EFM/Vs.
The bottom part of Figure 3 shows the main steps during post-processing. First, the info file, containing all information on the previously applied compressions, and the `mplrs`, respectively, `efmtool`'s output files are read. Since `mplrs`' output besides the calculated modes also includes the split reactions, as well as additional information, it requires additional steps before decompressions can be started. Therefore users need to specify whether `mplrs`' or `efmtool`'s output is to be decompressed. After parsing all input files a reverse decompression stack is built and each EFM, respectively EFV, is being decompressed one after the other in reverse order of the previously applied compressions until all modes are decompressed and written to a specified output. The resulting decompressed EFMs now contain all reactions as the original, uncompressed model and are ready for further analysis.

Algorithm 4 Pseudo-code snippet: *echelon*

```
function FINDS AND REMOVES REDUNDANCIES DUE TO CONSERVATIVE RELATIONS(N_{m,n})
    ▷ finds conservative relations and removes redundant metabolites from N
    ▷ N is the stoichiometric matrix that denotes m × n
    E_{i,j} ← reduced row echelon form of N
    k ← 0
    for i ∈ E do
        while i_k ≠ 1 do
            v ← v append k
            k ← k + 1
        end while
        k ← k + 1
    end for
    remove all m ∈ v from N
    return ΔN
end function
```

a) Run times of different geometric formulations (FC, PC and P) calculated with `mplrs`

b) Comparison of the amount of EFVs found in the cone shapes (FC, PC) versus in a general polyhedron (P)

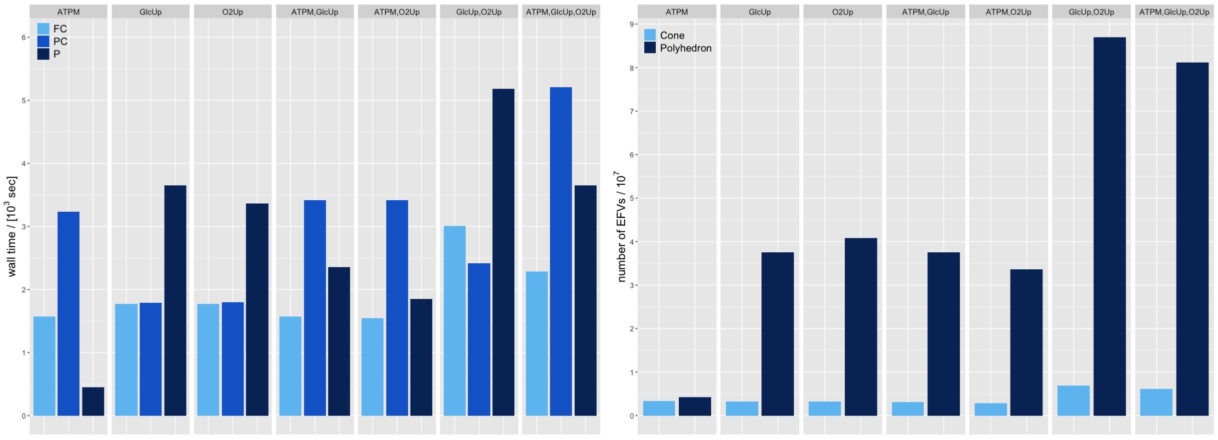

Figure S2 Figure S2 a shows the run times for the EColiCore2 [29] using different geometric formulations: flux cone FC (9) (in light blue, first column), polyhedral cone PC (7) (blue, second column) and general polyhedron P (5) (dark blue, third column); calculated with mplrs [1] with different reactions bounds applied, see Table 2. The y-axis shows the wall time in seconds (as 10 to the power of 3) and the headers indicate the applied reaction bounds. In 6 out of 7 cases using a cone results in faster run times compared to the general polyhedron. In a polyhedron all EFM/Vs get enumerated, however, some are listed multiple times [27]. A comparison of the amount of EFVs found in the cone shapes FC and PCcompared to the amount of EFVs found in the general polyhedron P is shown in Figures S2 b. The y-axis shows the number of EFVs as 10 to the power of 7. Interestingly, although the scenario with the reaction bound on ATPM contains 30% more results when calculated in a general polyhedron, it is still faster than the cone formulations.
